# Advancements in metabolic engineering: unlocking the potential of key organic acids for sustainable industrial applications

**DOI:** 10.3389/fbioe.2025.1556516

**Published:** 2025-03-11

**Authors:** Tengfei Wang, Han Xue, Hongling Liu, Haibo Yuan, Di Huang, Yi Jiang

**Affiliations:** ^1^ State Key Laboratory of Green Papermaking and Resource Recycling, Qilu University of Technology (Shandong Academy of Sciences), Jinan, Shandong, China; ^2^ Key Laboratory of Shandong Microbial Engineering, School of Bioengineering, Qilu University of Technology (Shandong Academy of Sciences), Jinan, Shandong, China

**Keywords:** pyruvate derivatives, metabolic engineering, microbial fermentation, carbon sources, organic acids

## Abstract

This review explores the advancements, application potential, and challenges of microbial metabolic engineering strategies for sustainable organic acid production. By integrating gene editing, pathway reconstruction, and dynamic regulation, microbial platforms have achieved enhanced biosynthesis of key organic acids such as pyruvate, lactic acid, and succinic acid. Strategies including by-product pathway knockout, key enzyme overexpression, and improved CO_2_ fixation have contributed to higher production efficiency. Additionally, utilizing non-food biomass sources, such as lignocellulose, algal feedstocks, and industrial waste, has reduced reliance on conventional carbon sources, supporting sustainability goals. However, challenges remain in substrate inhibition, purification complexity, and metabolic flux imbalances. Addressing these requires omics-driven metabolic optimization, stress-resistant strain development, and biorefinery integration. Future research should focus on system-level design to enhance cost-effectiveness and sustainability, advancing industrial bio-manufacturing of organic acids.

## Graphical Abstract

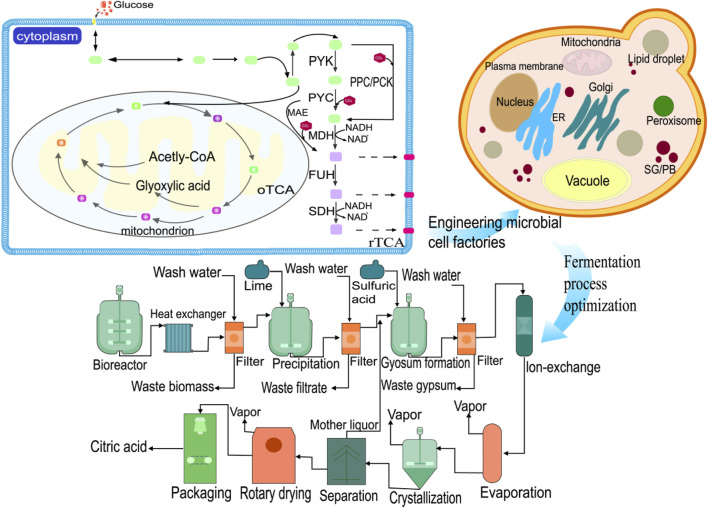

## 1 Introduction

Organic acids are essential in serving as intermediates for a wide range of industrial processes and find utility in various sectors including food and drink, pharmaceuticals, agriculture, and biochemistry. Among them, fumaric acid, lactic acid, butyric acid, succinic acid, malic acid, alpha-ketoglutaric acid, citric acid, and isocitric acid have received much attention due to their multifunctional effects and economic importance. Widely employed in the food sector, lactic acid a type of hydroxy acid serves to conserve freshness and amplify taste. It is also employed in pharmaceutical formulations and biotechnology to manufacture environmentally friendly plastics that are biodegradable. Recent progress in the domain of metabolic engineering has concentrated on enhancing biosynthetic routes and examining alternative carbon substrates to augment efficiency and yield, with gene suppression techniques emerging as a promising avenue for regulating enzyme activity.

Butyric acid plays a crucial role in food, pharmaceutical, and chemical industries, and is primarily generated through the microbial fermentation of substances like glucose and xylose. Current research is focused on utilizing low-cost alternative substrates and improving strain tolerance to increase yields and reduce costs. Succinate, an up-and-coming platform chemical, is being more and more generated through microbial fermentation using sustainable materials like lignocellulosic and citrus waste. The primary goal is to enhance strain effectiveness, fine-tune the fermentation procedure, and boost the use of pentose in order to minimize expenses and address environmental concerns. Its primary synthesis originates from the citric acid cycle, and the generation of α-ketoglutarate relies on essential catalysts like pyruvate oxidoreductase and α-ketoglutarate oxidoreductase. Metabolic engineering aims to optimize output by maximizing the performance of these pivotal enzymes and metabolic routes. Fumaric acid is produced through three metabolic pathways. TCA reduction cycle, TCA oxidation cycle and glyoxylic acid pathway. Each pathway has different efficiencies and limitations, and current research is focused on increasing yields and addressing inhibition in high-sugar environments. The primary method of production is through microbial fermentation, with microorganisms like *Aspergillus niger* being utilized to produce citric acid. Research advances include optimizing genetic engineering strategies, fermentation conditions, and adding precursors to improve yield and efficiency. Referred to as isocitric acid, this compound, while not as plentiful in the natural world, finds use in the fields of food production, pharmaceuticals, and biotechnology. The synthesis of it through the TCA cycle and glyoxylate cycle requires important enzymes like citrate synthase and isocitrate dehydrogenase. The direction of research work is genetic modification, fermentation optimization and cost effective utilization of raw materials. Malic acid is synthesized by TCA cycle and glyoxylic acid pathway, and is widely used in industry. Improving its production involves optimizing metabolic pathways and fermentation conditions to increase yield and reduce production costs.

These organic acids are key to driving a wide range of industrial applications, and continued research into microbial metabolic engineering is essential to increase production efficiency, reduce costs, and promote sustainable practices. In the pursuit of more environmentally friendly and economical options, it is crucial to focus on creating new types of microbial strains, improving fermentation methods, and investigating different raw materials in order to progress the manufacturing of these important organic acids.

In this paper, the metabolic engineering strategies and industrial applications of monocarboxylic organic acids, dicarboxylic organic acids and tricarboxylic organic acids are discussed according to the number of carboxylic groups. The important application prospect of organic acid metabolism in metabolic engineering is presented, and the current research progress of organic acid metabolism engineering is summarized and analyzed. In this paper, the production and significance of nine organic acids are introduced, with emphasis on the latest research trends and technical progress of microbial fermentation and metabolic engineering.

## 2 An organic acid with a carboxyl group

### 2.1 Pyruvate

Pyruvate and its related compounds are extensively used across healthcare, beauty, cosmetics, food, and multiple sectors. In manufacturing, pyruvate is mainly used for producing amino acids like L-tyrosine and L-tryptophan, as well as bioactive molecules such as N-acetylneuraminic acid (sialic acid), 3,4-dihydroxyphenylalanine (DOPA), and levodopa (Yuan et al., 2022).

**FIGURE 1:**
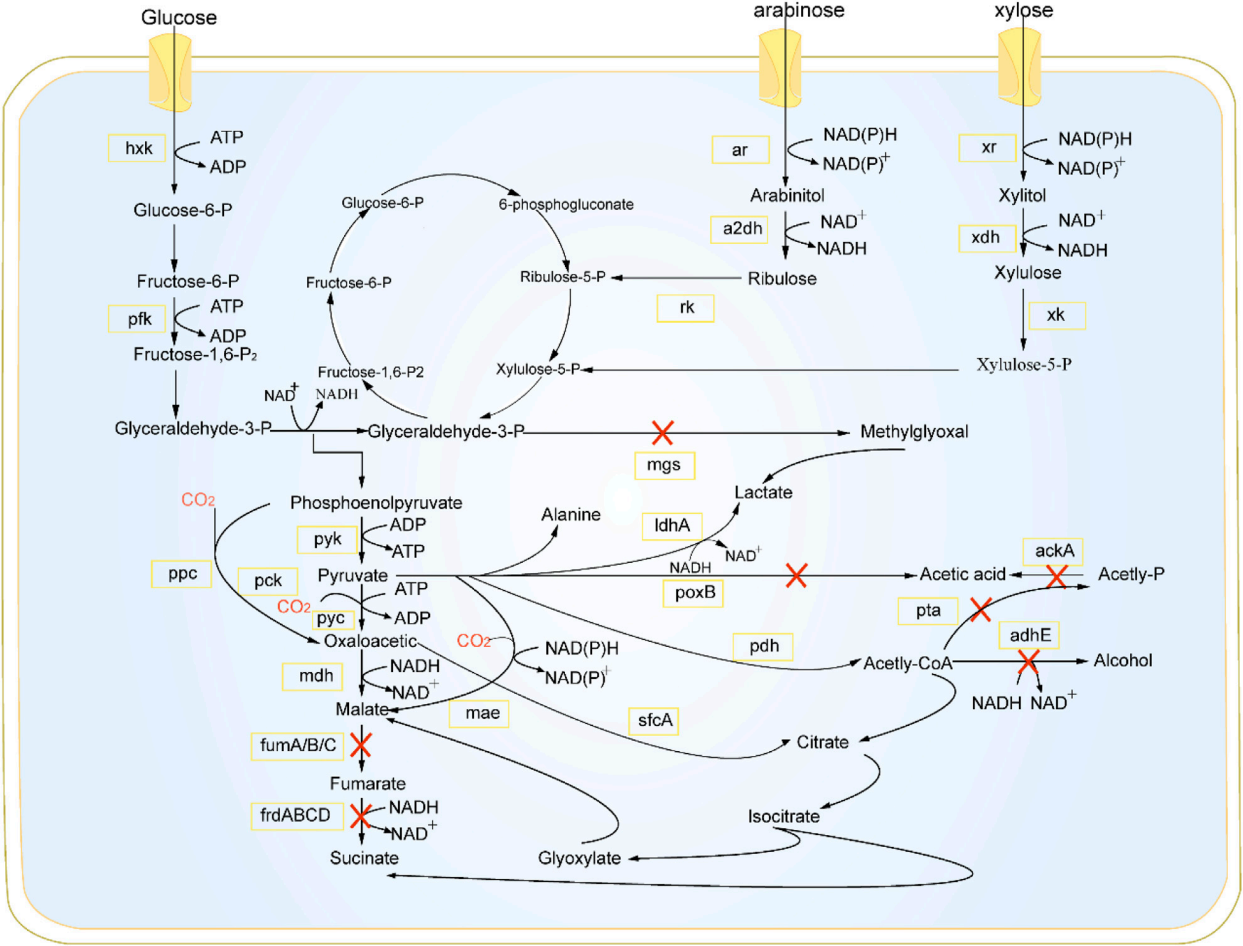
Pyruvate synthesis pathway. *hxk*, hexokinase; *pfk*, phosphofructokinase; *ar*, alcohol dehydrogenase; *a2dh*, aldehyde dehydrogenase; *rk*, kinase; *xr*, reductase; *mdh*, malate dehydrogenase; *xk*, xylulose kinase; *mgs*, methylglyoxal synthase; *pyk*, pyruvate kinase; *ppc*, pyruvate carboxylase; *pck*, phosphoenolpyruvate carboxykinase; *pyc*, pyruvate carboxylase; *fumA/B/C*, catalyzing the conversion of fumarate to malate; *frdABCD*, enzymes that reduce fumarate to succinate; *mae*, malic enzyme; *ldhA*, lactate dehydrogenase A; *poxB*, pyruvate oxidase; *pdh*, pyruvate dehydrogenase; *sfcA*, succinyl - CoA synthetase A; *ackA*, acetate kinase A; *pta*, phosphotransacetylase; *adhE*, aldehydedehydrogenase E.

To optimize metabolic engineering for microorganisms used in pyruvate biosynthesis, it is crucial to understand their metabolic regulatory mechanisms. At present, pyruvate is primarily generated via the glycolytic pathway of glucose metabolism occurring in the cytoplasm (Figure 1) (Wang et al., 2015). The glycolysis process ends with a final step, and the reactions of pyruvate can result in the production of various chemicals depending on oxygen levels (Wang et al., 2005). When oxygen is available, pyruvate is subjected to an oxidative decarboxylation process, resulting in the release of carbon dioxide and the formation of acetyl-CoA. Reactions between acetyl-CoA and oxaloacetate result in the production of citrate, allowing it to enter the TCA cycle. The efficacy of this process is contingent upon the activity of the pyruvate dehydrogenase complex. Conversely, devoid of oxygen, pyruvate is converted to α-acetolactate by the action of α-acetolactate synthase (*ALS*), which then leads to the 2,3-butanediol pathway or is transformed into acetate via pyruvate oxidase (*POX*). Simultaneously, surplus pyruvate is acted upon by pyruvate formate-lyase (*PFL*) to undergo conversion.

In the absence of oxygen, most bacteria, such as *Escherichia coli*, employ *ldh* to transform pyruvate into lactate (Xiong et al., 2024). In yeast, the enzyme pyruvate decarboxylase facilitates the transformation of pyruvate into acetaldehyde. Pyruvate has the ability to be converted into alanine by alanine transaminase (*ALT*) in both aerobic and anaerobic environments. The activity of key enzymes in the pyruvate biosynthesis pathway is influenced by feedback metabolites, and some enzymes are dynamically regulated by cofactors (Figure 1) (Cybulski et al., 2019). Zhang and his team disrupted the gene responsible for pyruvate decarboxylase (*KmPDC1*) and the glycerol-3-phosphate dehydrogenase gene (*KmGPD1*) in *Kluyveromyces marxianus* YZJ051. They then performed overexpression of *mth1* and its variants to improve the growth of KmPDC-null strains. Strain YZB053 was obtained through overexpressing the (*SsXYL2-ARS*) gene, resulting in an increase in the pentose phosphate pathway and xylitol dehydrogenase activity. This led to the production of 24.62 g/L of pyruvate from 80 g/L xylose at a temperature of 42°C, with a productivity rate of 0.51 g/L/h (Zhang et al., 2017). Cao et al. utilized a acid-resistant, pyruvate-tolerant strain of *Klebsiella oxytoca* PDL-0 and integrated the nox (NADH oxidase gene) into the *ldhD* locus to inhibit lactic acid production and regenerate NAD (Cao et al., 2020). Through deletion of the *cstA* and *yjiY* genes, the modified *K. oxytoca* PDL-YC strain achieved a yield of 71.0 g/L pyruvate from glucose (Cao et al., 2020). Wu et al. deleted several genes essential for by-product synthesis in *Vibrio natriegens*. Through the expression of *ppc* gene, 54.22 g/L of pyruvate was produced to balance cell growth and pyruvate synthesis (Wu et al., 2023).

Currently, *E. coli* and yeast are the primary microorganisms used for pyruvate fermentation (Wang et al., 2022). These organisms are favored for their capacity to gather and release substantial amounts of pyruvate, their quick growth, efficient conversion rates, and non-harmful nature to humans and animals. Novel strategies in metabolic engineering are increasingly focusing on suppressing gene expression rather than removing genes entirely. One example includes the suppression of the *aceE* gene, which affects pyruvate dehydrogenase and retains some level of activity in this enzyme, enabling glucose to be the sole carbon source (Moxley et al., 2021). Current research directions explore the use of alternative substrates, including whey, alginate (Kawai et al., 2014), mannitol (Yoshida et al., 2015), and lactic acid (Gao et al., 2010), to produce pyruvate. These efforts indicate that future studies will likely prioritize the use of low-cost and unconventional carbon sources in producing pyruvate (Gao et al., 2010).

### 2.2 Lactic acid

Lactic acid, a prevalent hydroxy acid in the natural world, engages in numerous reactions that yield an array of its derivatives (Ren et al., 2022). It is particularly noted for its role in the creation of polylactic acid, which stands as the most valuable biodegradable plastic raw material in commerce (Abdel-Rahman et al., 2013). This chemical entity is pivotal in the manufacture of eco-friendly polymers, oxygenated compounds, sustainable solvents, and plant growth promoters (Nwamba et al., 2021).

At present, advancements in lactic acid manufacturing are contingent upon the utilization of metabolic engineering and conventional fermentation methods (Eş et al., 2018). In the cytoplasm of eukaryotic cells, glycolysis breaks down a single glucose molecule into two pyruvate molecules, which are subsequently transformed into lactic acid through the lactic fermentation pathway by lactate dehydrogenase (Ren et al., 2022). Lactic acid fermentation in prokaryotes is divided into two types. Homologous fermentation is similar to lactic acid fermentation in eukaryotes, both of which produce L-lactic acid. Opposite-sex fermentation will produce D-lactic acid, but also ethanol, acetic acid and other by-products (Tian et al., 2021a).

Traditional metabolic engineering methods focus on pathway redirection and heterologous gene expression. Following the consumption of glucose by certain host microorganisms, they may utilize the resulting end product for growth. By disrupting the genes responsible for D-lactate dehydrogenase and monocarboxylate transporter, the utilization of D-lactic acid can be effectively eradicated. Furthermore, specific microorganisms possess the capability to break down heterozygosis and produce substances such as ethanol. These byproducts can be removed by inactivating the *gpd1* and *gpd2* genes, which encode glycerol-3-phosphate dehydrogenase (Liu et al., 2023b). The low acid tolerance of host cells in LA producers is not favorable for industrial production (Liu et al., 2023b). Therefore, the use of acid-resistant strains for metabolic engineering is a good method. Liu et al. utilized a *S. cerevisiae* TAM strain that demonstrated tolerance to pH 2.4 as the initial strain. Subsequent adjustments in energy supply and redox balancing led to an increase in L-LA titer, reaching 72.7 g/L during shake-flask fermentation without the use of a neutralizer, with a yield of 0.66 g/L/h (Liu et al., 2023b). In another example, heterologous *ldh* gene was expressed in acid-resistant *S. cerevisiae* CEN. PK2 and ethanol-producing pathways *pdc1* and *adh1* were knocked out. The yield of *E. coli* acetyl-CoA synthesis pathway reached 142 g/L (Juodeikiene et al., 2016).

In recent years, CRISPR-Cas9 technology has been used to successfully develop high optical purity of *Lactobacillus paraceo* strains, in order to achieve an optical purity of over 99.1% for L-lactic acid in the fermentation liquid (Tian et al., 2021b). At the same time, low-cost green substrates such as lignocellulose, glycerin (Jodłowski and Strzelec, 2021), matrine residue (Ma et al., 2020), municipal household waste (Acedos et al., 2022) and sugarcane molasses (Sun et al., 2019) were developed. This suggests that future lactic acid production research will focus on improving optical purity and developing low-cost substrates.

### 2.3 Butyric acid

Butyric acid is extensively employed in the creation of industrial chemicals, food items, pharmaceuticals, and additives for animal feed (Fu et al., 2022). It holds the capacity to act as a precursor for specific cellulosic organic acid esters utilized in the coating industry, providing superior protection against light, heat, and moisture (Jiang et al., 2018). Within the food and beverage sector, butyric acid is used to intensify buttery flavors, often supplemented to enhance the taste of fruits, and is a crucial component in the manufacture of aromatic compounds for flavoring (Fu et al., 2017).

The microbiological research primarily focuses on several Gram-positive bacteria, including *Butyribacterium*, *Butyrivibrio*, *Clostridium*, and *Eubacterium*. Glucose is converted into pyruvate in the cytosol through the glycolytic pathway, which is then decarboxylated by pyruvate-ferredoxin oxidoreductase to generate acetyl-CoA (Guo et al., 2024).

Although the cost of fermentation to produce butyric acid is no advantage compared to chemical synthesis, it is necessary in industries such as beauty (Mariën et al., 2023). In a research experiment, the increased expression of acid-resistant Class I heat shock protein (hsg) had significant effects on strains in conjunction with the increased expression of *dnaK* and *groE* operons. The strain’s acid tolerance was greatly improved by *groESL*, while *dnaK* had a detrimental effect on the strain’s ability to tolerate acid. Compared with the control group, the modified strain increased by 15%, and the titer reached 52.2 g/L (Suo et al., 2017). Part of the phosphotransacetylase gene (*pta*) from *C. tyrosine* was integrated into *C. butyricum* by homologous substitution, which disrupted the acetic acid formation pathway. Part of the *pta* (phosphotransacetylase gene) from *Clostridium tyrosine* was integrated into *Clostridium butyricum* by homologous substitution, which disrupted the acetic acid formation pathway. Compared with the original strain, the modified strain had a titer of 32.5 g/L (Guo et al., 2024). Due to the shortage of tools and the difficulty of transformation in the genetic engineering of *Clostridium difficile*. In a particular research, the native *fadR*, *aceF*, *ldhA*, and pta genes in *E. coli* were inactivated, resulting in a modified strain that yielded 3.3 g/L of butyric acid in a medium containing 10 g/L of glucose (Seregina et al., 2010).

Due to the presence of carbon decomposition metabolite inhibition (CCR), several *clostridium* bacteria used for butyric acid fermentation are strongly inhibited by glucose while utilizing xylose. In one study, replacement *xylA* (xylose isomerase gene), *xylB* (xylokinase gene) and *xyylt* (xylose proton homology gene) were co-expressed in *Clostridium tyrobutyricum*. The modified strain can produce butyric acid from xylose and glucose at the same time, and the final titer reaches 46.4 g/L (Fu et al., 2017).

The study of butyric acid fermentation through traditional strain breeding and metabolic engineering strategies will be the focus. Currently, most studies use agricultural derivatives such as glucose and xylose as substrates, which are not only expensive but also compete with the food industry, which is not only economically but also resource-wise unsustainable. However, the use of cheap lignocellulosic substrates would result in high pretreatment costs and inhibition of the microbial strain (Kelbert et al., 2024). The high cost of production is due to the low yield of butyric acid and the difficulties in separating and purifying the co-product, acetic acid (Wang et al., 2019). In the future, research on butyric acid can be conducted by adopting alternative inexpensive substrates to avoid excessive substrate costs and to avoid competition with the food industry (Guo et al., 2024; Mariën et al., 2023). The utilization of underutilized substrates in industrial and agricultural sectors for fermentation to produce butyric acid has become a focus of interest for researchers (Wang et al., 2019).

## 3 Organic acids with two carboxyl groups

### 3.1 Succinic acid

It is anticipated that succinic acid will soon become one of the most significant platform chemicals, alongside malic acid. Furthermore, it will serve as a plasticizer within the food industry. In recent years, various engineering approaches have been established to produce succinic acid through microbial fermentation. Due to their ability to withstand low pH levels, microorganisms like *Saccharomyces cerevisiae* (Liu et al., 2021) and *E. coli* are being considered (Chiang et al., 2021), as they can play a crucial role in reducing downstream expenses.

**FIGURE 2:**
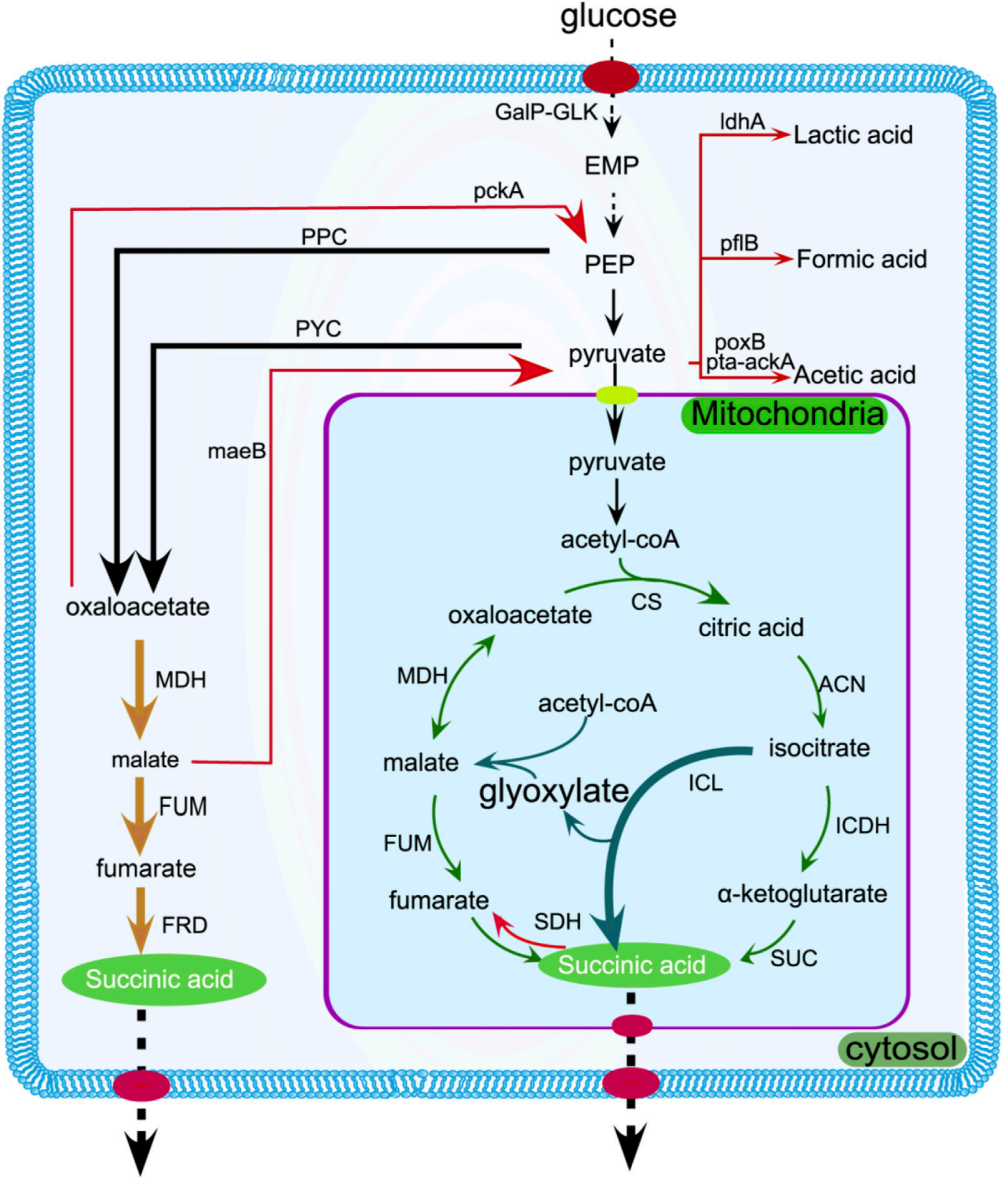
Succinic acid is produced in the cytoplasm and mitochondria through the rTCA pathway (yellow arrow) and oTCA pathway (green arrow), respectively. The enzymes can be enhanced to increase metabolic flux, as indicated by the bold arrows. The glyoxylate pathway is represented by the dark green arrow, while gene suppression or deletion is denoted by the red arrow. Transmembrane transport is shown by dotted arrows. *GalP-GLK*,It refers to a transport system in *Escherichia coli* (*E. coli*) that involves two components: galactose penetrase (*GalP*) and glucokinase (*GlK*); *pflB,* pyruvate formate lyase; *cs*, citrate synthetase; *ldhA*, lactate dehydrogenase A; *can*, aconitase; *sdh*, succinate dehydrogenase; *frd,* fumarate reductase.

At present, there are three distinct methods for catalyzing the synthesis of succinic acid: oTCA, rTCA, and the glyoxylate pathway. The rTCA pathway to form succinic acid is currently a hot spot, and the ability to fix a molecule of carbon dioxide is very consistent with the current environmental protection concept. The yield of succinic acid can be improved by boosting the activity of pivotal enzymes within the TCA cycle, such as succinate dehydrogenase (*SDH*), fumarate reductase (*FRD*), and malate dehydrogenase (*MDH*) (Ahn et al., 2020). Intracellular succinic acid needs to be transported outside the cell as soon as possible, avoiding key enzyme activity that affects intracellular pH and the synthesis pathway. The succinic acid transporter SucE was found by Li et al. to have a titer of 68.66 g/L encoded by NCgl2130 (Figure 2) (Li et al., 2017).

The main carbon source of commercially produced succinic acid is a variety of common sugars, such as sucrose (Lee et al., 2016) and glucose (Babaei et al., 2019). Serving as a crucial source of energy for the metabolic processes of living organisms, glucose also generates numerous chemical compounds and essential substances for conversion during the catabolic process (Figure 2). It is the most commonly used carbon source for producing chemicals derived from biological sources. Reaching elevated levels of succinic acid via the glucose metabolism is equally attainable with the efficiency of succinic acid (Ferone et al., 2019). The focus on using sustainable raw materials for producing succinic acid is increasing, with a goal of capturing carbon and reaching carbon neutrality. In order to make use of lignocellulose, a metabolically engineered strain of *E. coli* Suc260 (pTbglA) was used to overexpress the *bglA* gene, which encodes β-glucosidase. This led to the generation of 25.06 g/L of succinic acid using cellobiose as the carbon source (Dong et al., 2017). Related to the environmental concept, it is reported that citrus peel waste is distilled and extracted by dilute acid hydrolysis to obtain pectin (30.53%) and essential oil (0.43%, including 17 compounds, mainly D-limonene). At the same time, 22.4 g/L of succinic acid was produced through fermentation (Patsalou et al., 2020). Succinic acid can be produced from residual biomass hydrolysates obtained by direct biomass esterification from microalgae (Sorokina et al., 2020). Bagasse (Shaji et al., 2021) can also be used as a carbon source for succinic acid biosynthesis. In the process of hydrolysis, an acid or base is commonly employed to eliminate lignin and break down cellulose and hemicellulose, resulting in the production of cellulase and xylanase for saccharification. However, the concentration of succinic acid remains comparatively low, ranging from 20 to 60 g/L, due to the high concentration of pentoses in the hydrolysates of lignocellulosic biomass like olive pits, bagasse, and straw (Lee et al., 2022).

Although biobased processes are more energy efficient and have less environmental impact compared to petrochemical processes, it is important to acknowledge that they also come with their own set of limitations. Despite lower raw material costs, the expensive, laborious, and intricate downstream processes make it less accessible. Much of the downstream cost comes from crystallizing succinic acid and removing various impurities or byproducts by lowering the pH of the fermentation solution. Given this context, the current focus of research is on enhancing the strain’s production efficiency, refining the fermentation process, and minimizing raw material expenses (Thangarasu et al., 2018).

### 3.2 L-malic acid

L- Malate, also known as 2-hydroxysuccinic acid, molecular formula C_4_H_6_O_5_, its powder is white crystalline, easily soluble in water and ethanol and other solvents (Wei et al., 2021).

Malic acid, recognized for its distinctive and agreeable taste, is extensively employed in the food and beverage sectors to boost flavor profiles. Additionally, it plays a role in the production of unsaturated polyester resins and coatings (Ding and Ye, 2023). Sunitinib malate, which acts as a tyrosine kinase inhibitor, exhibits both anti-angiogenic and anti-tumor properties and is approved by the FDA for treating renal cell carcinoma and gastrointestinal stromal tumor (Wu et al., 2022).

In the past 20 years, different strains have been created to produce malic acid, with each strain having its own set of strengths and weaknesses. For example, *E. coli*, which has been widely studied among bacteria, has rapid growth and reproduction and simple genetic manipulation, but it is non-food safe, has low substrate tolerance concentration and limited acid production capacity (Zhu et al., 2020). *S. cerevisiae* can withstand high-sugar and high-acid fermentation environment (Kang et al., 2021), but the reported malic acid production intensity is low, and the level of heteroacid is high. So far, the highest yield of malic acid production strain reported is *Ustilago trichophora* (Zambanini et al., 2017), but it is a plant pathogen, and the fermentation cycle is about 10–12 days. Some natural strains, such as *Aspergillus oryzae* (Ji et al., 2021), can accumulate a large amount of malic acid when cultured with high glucose concentration, appropriate nitrogen source, inorganic salt and CaCO_3_.

**FIGURE 3:**
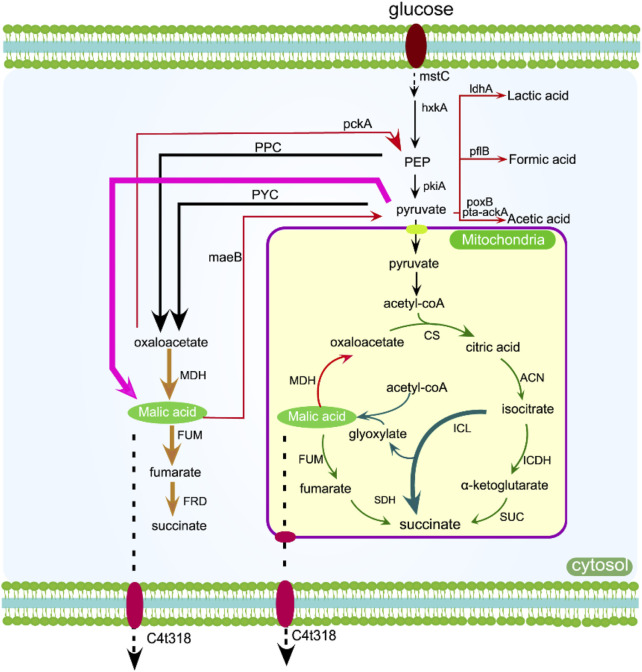
The production of malic acid can be increased by overexpressing enzymes in the rTCA pathway (yellow arrow), oTCA pathway (green arrow), glyoxylic acid pathway (dark green arrow), and one-step pathway (pink arrow). Genes that may inhibit or delete the metabolic flux are indicated by red arrows, while transmembrane transport is shown with dotted arrows. C4t318, C4-dicarboxylate transporter; *mstC*, glucose low-affinity transporter; *hxkA*, hexokinase A; *pckA*, phosphoenolpyruvate carboxykinase A; *mdh*, malate dehydrogenase.

The rTCA pathway is the most efficient among L-malic acid metabolic pathways in terms of carbon conversion rate. Therefore, enhancing or building the rTCA pathway has consistently been the favored approach for metabolic engineering aimed at increasing L-malic acid production, as seen in Figure 3 (Wei et al., 2021). The rTCA pathway produces oxaloacetic acid by fixing CO_2_, which is further converted into malic acid, and has high carbon efficiency (Wu et al., 2022). In addition, the combination expression of L-malic acid transporters, the directed evolution of key enzymes, and the regulation of coenzyme regeneration are also important methods to improve the yield of L-malic acid (Jiang et al., 2021). The production process initiates with the carboxylation of phosphoenolpyruvate or pyruvate and the by-products of glycolysis. Consequently, managing the metabolic fluxes of glycolysis, the reductive tricarboxylic acid (rTCA) cycle, and CO_2_ fixation is crucial for the efficient biosynthesis of malic acid, which is shown in Figure 3 (Chen et al., 2023a).

Xu et al. developed a genome editing system, Cre-loxP, to modify the metabolic flux of organic-producing strains for malic acid production. They utilized this system to delete *oahA* and integrate *pyc*, *mdh3*, and *c4t318* overexpressed in *A. niger* in order to enhance the metabolic flux of the rTCA pathway and increase malic acid output. After 9 days of fed-batch fermentation, they achieved a titer of 201.24 g/L (Xu et al., 2020). In the rTCA pathway of filamentous fungi, malate dehydrogenase is dependent on the coenzyme NADH in the cytoplasm. To address this limitation, *E. coli stha* is an energy-independent FAD-containing enzyme. The catalyzed reversible reaction of NAD (H) and NADP (H) leads to a significant increase in the production of L-malic acid in *A. niger* (Yang et al., 2024). Chen et al. achieved a titer of 235.8 g/L MA from the final engineered strain by overexpressing *trmae1*, a natural MA transporter gene in Trichoderma reesei. The yield was 1.48 mol MA per mole of glucose and the productivity reached 1.23 g/L/h (Chen et al., 2023b). This represents the maximum titer that has been documented. In general, improving the metabolic flux and rTCA pathways within glycolysis is an optimal approach for boosting malic acid output.

The employment of microbial metabolism to convert it into valuable products has emerged as a prominent area in the biological field lately (Wei et al., 2021). Although L-malic acid fermentation technology’s industrialization has been reported, its production rate and purity are not high, subsequent separation and purification are challenging, and costs are significant. Studies on filamentous fungal fermentation for L-malic acid production have only reached laboratory stages without any industrialization yet (Li et al., 2023).

### 3.3 Alpha-ketoglutaric acid

In the field of medicine, it acts as an important precursor for creating different medications and can be used to generate antioxidants (Zhang et al., 2020), thus improving antioxidant levels (Li et al., 2016). Within the food industry, α-ketoglutaric acid functions as a nutritional component in sports beverages, offering energy and preventing ammonia toxicity while also serving as an antimicrobial agent (Legendre et al., 2020).

Reproduction of alpha-ketoglutarate is achievable through chemical synthesis, enzymatic processes, and microbial fermentation. While the industrial production currently relies on chemical synthesis, this method presents challenges such as a complex process, difficult separation, and environmental pollution (Liu et al., 2023b). In contrast, microbial fermentation offers advantages including low cost, high efficiency, and environmental friendliness, making it a promising option for industrial production (Yovkova et al., 2014).

The biosynthesis pathway of α-KG was improved in *E. coli* by reorganizing the TCA cycle to increase pyruvate production, and additional improvements were achieved by adjusting the levels of gene expression. Furthermore, enhancing the supply of acetyl-CoA can alleviate the constraints in the synthesis of α-ketoglutarate (α-KG) (Chen et al., 2020). The metabolic intermediate α-KG is essential for the Tricarboxylic acid cycle (TCA cycle) in microbial cells (Chen et al., 2020). Carbon source materials from the environment are transported into cells and converted to pyruvate through glycolysis. The tricarboxylic acid cycle utilizes carbon sources with the help of pyruvate dehydrogenase complex, citrate synthase, aconitase, and isocitrate dehydrogenase to produce α-KG. Subsequently, α-KG is further metabolized into succinyl-CoA by α-ketoglutarate dehydrogenase (*KGDH*), accompanied by electron transfer and energy generation. This process provides both carbon source material and energy for cell growth and reproduction. Furthermore, α-KG participates in nitrogen metabolism by transamination to form L-glutamate, thus serving as a vital metabolic intermediate that links carbon metabolism with nitrogen metabolism (Song et al., 2016).

A variety of metabolic engineering tactics have been devised for the biosynthesis of α-ketoglutaric acid, employing *Clostridium glutamicum* as the production host (Tenhaef et al., 2021). Through overexpression of *pyc* or *pdh* complexes (Yin et al., 2012), the carbon pathway of pyruvate into the TCA cycle is enhanced to produce α-KG. Nevertheless, the build-up of secondary substances like pyruvate poses challenges and expenses for the subsequent separation process (Yin et al., 2012). Increased expression of genes that encode glycerol kinase, methylcitrate synthase, and transporters for organic acids in the mitochondria. Alpha-KG of 53.1 g/L was obtained with a productivity of 0.35 g/L/h (Tomaszewska-Hetman et al., 2021). The overexpression of NADP-dependent isocitrate dehydrogenase (*IDH*) and *pyc1* in fatty acid degrading yeast significantly enhanced the synthesis of α-ketoglutaric acid, resulting in a remarkable increase in the yield to 186 g/L after fermentation for 117 h. Bovine et al. successfully expressed L-glutamate oxidase (*LGOX* ^*Str*^) and catalase (*KatG* ^*Esc*^) from *Streptomyces virusosporus* R111 in *E. coli* H736. Through the addition of sfGFP tags, they were able to anchor L-glutamate oxidase (*KatG* ^*Esc*^) and catalase (*KatG* ^*Esc*^) to the outer membrane of *E. coli* cells, allowing for one-step whole-cell catalysis of alpha-ketoglutaric acid with a conversion efficiency of up to 75% (Niu et al., 2024).

Microbial fermentation can significantly reduce the overall production cost of α-KG. To achieve this goal, subsequent improvements will primarily focus on fine-tuning the metabolic pathway by lowering nodes in the tricarboxylic acid cycle and dehydrogenases such as *kgdh* and *icl*, while increasing the activity of pyruvate dehydrogenase (*PDH*) to balance the host cell metabolism, enhance production efficiency, and design optimized cell phenotypes to enhance substrate utilization and environmental tolerance (Zhou et al., 2023).

### 3.4 Fumaric acid

Commonly known as an essential substance, fumaric acid is of significant value in a range of sectors such as food and beverage, cleaning products, animal nutrition, pharmaceuticals, and other industrial goods (Sebastian et al., 2019). With the increasing emphasis on protecting the environment and promoting sustainable development, new challenges and opportunities are emerging for the production of fumaric acid. The traditional petrochemical route has problems such as high energy consumption and high pollution emissions. Therefore, more and more companies are turning to “green production” and environmental protection technologies. During the implementation of “green production,” there is a strong focus on utilizing inexpensive raw materials for the biotechnological production of fumaric acid (Sebastian et al., 2021). By utilizing microorganisms or other biological pathways containing or capable of obtaining fumaric acid raw materials, combined with modern process technology for extraction and purification can effectively reduce costs and reduce reliance on traditional resources. At the same time, this method also has good environmental friendliness, meeting the urgent needs of today’s society for sustainable development and resource conservation (Sebastian et al., 2019).

Fumaric acid is generated via three distinct metabolic routes. The reductive arm of the citric acid cycle is pivotal in cellular energy metabolism, transforming pyruvate into oxaloacetate and enabling ATP production. This metabolic sequence boasts a theoretical maximum yield of 2 moles of ATP per mole of glucose and necessitates ATP and CO_2_ for the carboxylation of pyruvic acid into oxaloacetic acid. Oxaloacetic acid is then converted into fumarate via malate dehydrogenase (*MDH*) catalyzed decarboxylation reaction, which includes an NADH reduction step (Guo et al., 2020).

The alternative route includes the oxidative TCA cycle, which is essential for producing fumarate. In this process, pyruvate is initially transformed into acetyl-CoA through the pyruvate dehydrogenase complex. Acetyl-CoA is involved in the tricarboxylic acid cycle, leading to the formation of succinate. Subsequently, succinate is acted upon by succinate dehydrogenase (*SDH*) to form fumarate, achieving a theoretical maximum yield of 1 mole per mole of glucose due to the release of CO_2_ during the reaction (Xu et al., 2013).

The potential application of the glyoxylate shunt in the production of fumaric acid is currently being evaluated. Isocitric acid, produced in the TCA cycle, is transformed into succinic acid and glyoxylic acid through the activity of isocitrate lyase (Johannsen et al., 2018). Subsequently, malic acid is formed through the reaction of glyoxylic acid and acetyl-CoA with the help of malate synthase. Despite having a lower potential yield (1 mole/mole glucose) compared to the reductive TCA cycle, the glyoxylate pathway demonstrates potential due to its more efficient metabolic pathway (Guo et al., 2020). However, in high-sugar environments, this key metabolic pathway is strongly inhibited and difficult to activate when glucose is used as a substrate. The suppression is caused by phosphoenolpyruvate (PEP), a product of glucose metabolism that acts as an inhibitor for isocitrate lyase (Sebastian et al., 2019).

At present, the main research object of fumaric acid production of *A*. *oryzae* has increased the yield of fumaric acid by 26% through overexpression of *ppc* (Li et al., 2014). A prevalent approach is to focus on *E. coli* to amplify the expression of genes associated with the citric acid cycle, like those for fumarase (*fum*) and fumarate reductase (*frd*). Moreover, augmenting the flow through the glyoxylate cycle can significantly boost the production of fumaric acid. Additionally, interrupting the primary side-product formation pathway is also a widely adopted strategy (Xu et al., 2013).

In the manufacture of industrial organic acids, the cost of substrates accounts for roughly 30%–40% of total costs (Jimenez-Quero et al., 2020). While glucose is commonly used as a carbon source for fumaric acid production, alternative sources including glycerol, xylose, and sucrose have been explored as well. However, these alternative carbon sources generally have lower fermentation efficiency compared to glucose due to the need for additional pathways to connect them to glycolysis and the TCA cycle. In light of growing economic and environmental pressures, there is a growing interest in exploring renewable raw materials that are abundant in starch or lignocellulose as potential alternatives (Ilica et al., 2019).

The future focus of metabolic engineering for fumaric acid production should prioritize global regulation rather than solely concentrating on individual genes or specific pathways. Novel techniques such as mitochondrial manipulation, scaffold modification, and cofactor adjustment will provide valuable knowledge for improving the procedure (Pairazaman et al., 2024). Utilizing waste biomass or co-substrate fermentation may present a more economical approach to enhancing the feasibility of biological production procedures. Additionally, optimization strategies should not only aim to increase yields at the laboratory scale but also consider industrial-scale fermentation feasibility to enhance economic competitiveness (Deng and Aita, 2018).

## 4 An organic acid with three carboxyl groups

### 4.1 Citric acid

Citric acid is mainly extracted from various sources of carbohydrates such as molasses and starch-based culture media, and produced by deep fermentation of *A. niger* (Rakicka et al., 2019). In order to facilitate extensive production, it is essential to guarantee that the manufacturing process is eco-friendly by making use of readily available and cost-effective agro-industrial residues, all while sustaining high levels of output (Mores et al., 2021).

*A.niger* is commonly used as a microorganism in the manufacturing process of citric acid. By means of fermentation, *A. niger* transforms glucose or alternative carbon sources into citric acid through the EMP (glycolytic pathway) and TCA cycles (Angumeenal and Venkappayya, 2013). Crucial enzymes involved in this process include Citrate synthase, which aids in the formation of citric acid from acetyl-CoA and oxaloacetate, and ATP-citrate lyase, which plays a vital role in citric acid synthesis (Ozdal and Kurbanoglu, 2018). *Yarrowia lipolytica* is another significant producer of citric acid, particularly when utilizing raw materials such as petroleum and ethanol. Similar to *A. niger*, *Y. lipolytica* converts carbon sources into citric acid through a comparable metabolic pathway with high conversion efficiency (Carsanba et al., 2019). In industrial production settings, various approaches are employed to enhance the yield and efficiency of citric acid production. These methods include genetic engineering to strengthen the biosynthetic pathway of citric acid, optimization of fermentation conditions, and inhibition or bypassing of metabolic pathways unrelated to or competing with the accumulation of citric acid (Yuzbasheva et al., 2019). For instance, genetic engineering can be used to enhance the expression of citrate synthase or optimize fermentation conditions such as pH level, temperature control, oxygen supply regulation for improved production efficiency (Angumeenal and Venkappayya, 2013).

**FIGURE 4:**
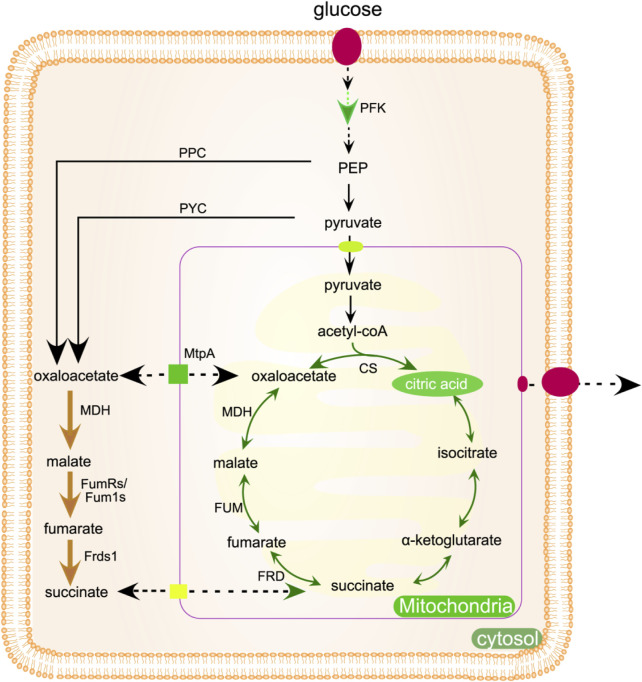
Metabolic engineering methods can be used to improve the production of citric acid, which is produced in mitochondria. Enzymes indicated by bold arrows may be increased or altered to boost metabolic flux. The rTCA pathway is represented by the yellow bold arrow, while the *PFK* can be modified to alleviate feedback inhibition as shown by the green bold arrow. Dotted arrows indicate transmembrane transport. *Pfk*, 6-phosphofructokinase; MtpA, mitochondrial transport protein A; *mdh*, malate dehydrogenase; *FumRs*, fumarate reductase; *Fum1s*, fumarase; *ppc*, pyruvate carboxylase; *pckA*, phosphoenolpyruvate carboxykinase A; *Frds1*, fumarate reductase d1; *pyc*, pyruvate carboxylase.

The production of citric acid can be increased by knocking out the by-product generating gene. For example, when corn starch is used as carbon source, deletion of isomaltose synthesis gene A-glucosidase coding gene *agdA* can effectively reduce isomaltose concentration and increase citric acid production (Wang et al., 2016). Enhancing the production of citric acid via precursor engineering can be achieved by supplementing the reaction with acetyl-CoA and oxaloacetic acid, the precursors necessary for citric acid synthesis. Several enzymatic systems contribute to the generation of acetyl-CoA, including pyruvate dehydrogenase (*PDH*) and cytoplasmic acetyl-CoA synthetase (*ACS*) (Kamzolova, 2023). Overexpression of *mdh2* (malate dehydrogenase), fumarate reductase (*FumR*) and fumarate reductase (*Frds1*) can increase the synthesis of oxaloacetic acid, shown in Figure 4. The strain that inhibited the engineered mutant phosphofructokinase *pfk1* by feedback produced 70% more citric acid than the control strain (Figure 4) (Hu et al., 2019). Murchitine synthetase gene (*chsC*) interferes with RNA. Following the silencing of the *chsC* gene, *A. niger* mutant strains form compact mycelial pellets, which reduce the viscosity of the medium and enhance the mass transfer of oxygen. Consequently, this leads to a 42.6% increase in citric acid yield compared to the wild-type strain (Hu et al., 2019).

The selection of carbon source greatly influences citric acid production, with monosaccharides and disaccharides being the most suitable options for carbon sources (Kadooka et al., 2020). The decreased enzyme activity in the fermentation medium is responsible for the slow rate of polysaccharide hydrolysis, leading to changes in pH value (Ajala et al., 2020). Sucrose is superior to glucose, fructose and lactose in increasing citric acid yield. Sucrose can be rapidly hydrolyzed at low pH. The presence of nitrogen has been shown to significantly impact the synthesis of citric acid, as nitrogen is essential for both cellular protein formation and metabolism (Morgunov et al., 2020). The synthesis of citric acid and fungal growth were influenced by the type of nitrogen source. It is essential to restrict the supply of nitrogen, as concentrations exceeding 0.25% result in urea accumulation and a reduction in citric acid production. High nitrogen concentration will increase carbon source consumption and fungal growth while reducing citric acid production (Rzechonek et al., 2019).

The presence of oxygen is crucial for the biological synthesis of citric acid, as it significantly influences the production process (Zhang et al., 2022). Receiving varying levels of aeration will negatively impact the fermentation process and overall production output. With an elevated aeration rate, the partial pressure of dissolved carbon dioxide in the medium was found to decrease. The substrate for pyruvate carboxylase (*PC*) is carbon dioxide, which serves as a replacement for the oxaloacetic acid needed by citrate synthase (*CS*). Pyruvate decarboxylation is catalyzed by pyruvate decarboxylase (*PDC*) to produce carbon dioxide, but extreme aeration conditions will cause certain losses (Figure 4). Increased carbon dioxide levels can damage final biomass and citric acid concentrations.

Currently, microbial fermentation is the dominant method for producing citric acid, and it is highly developed. However, there is still room for improvement in the purification process downstream. In the future, with the continuous progress of science and technology, it is expected to shift from mature production technology to new cheap raw materials and new recovery methods of citric acid. This will greatly promote the improvement of resource utilization efficiency in the production process of citric acid, and also have a positive significance for environmental protection (Rzechonek et al., 2019).

However, although the technology is quite mature at this stage, it still needs to continue in-depth research to reach a higher level. For example, in the process of microbial fermentation, how to improve the production of citric acid, reduce energy consumption, reduce waste emissions and other aspects need to continue to explore and improve. At the same time, in the application of new materials and new equipment also need to continue to innovate and improve.

In short, although the microbial fermentation production of citric acid has made remarkable achievements, in the future there is still a need to continue to promote scientific research and practice in related fields, in order to achieve a more sustainable, efficient and environmentally friendly production of citric acid.

### 4.2 Isocitric acid

Isocitric acid, a less common isomer of citric acid, has various industrial applications despite its lower natural abundance (Rzechonek et al., 2019). It can be used as an acid agent in food and beverages, enhancing the taste of food and extending the shelf life. In pharmaceutical manufacturing, it can be used in drug synthesis or as an auxiliary ingredient in some drugs, such as pH regulators or stabilizers (Kamzolova et al., 2016). In addition, it can also be used as a detergent ingredient to remove stains and improve cleaning effect; In the field of biotechnology, it can be used as metabolic intermediates in the process of microbial fermentation to produce other biological chemicals (Kamzolova et al., 2021). In the agricultural field, it can be used as a plant growth regulator or some pesticide formulations. Simultaneously, it has the potential to serve as a starting material for the production of various organic compounds in the chemical industry and holds significance in research, such as its use as a possible precursor material for biofuel production (Kamzolova et al., 2016).

The isocitrate molecule has four different forms, but only one of them, threo-DS-isocitric acid, is important for the TCA cycle in aerobic organisms (Kamzolova et al., 2018). In recent studies, ICA has been examined for its potential as a natural agent for prevention and treatment. Specifically, its effectiveness in treating iron deficiency anemia and in absorbing blood clots has been documented (Kamzolova and Morgunov 2019). Trimethylcitrate is currently being investigated as a novel treatment for Parkinson’s disease associated with *dj-1* gene dysfunction (Kamzolova et al., 2018). It has been hypothesized that membrane-permeable trimethyl isocitrate prevents dopamine production from disrupting DNA structure in mitochondrial neurons. ICA, which exists in the form of lactones with chiral properties, has great potential in the chemical and pharmaceutical industries (Morgunov et al., 2020).

The synthesis of isocitric acid is a complex biochemical process, mainly completed through the TCA cycle and glyoxylate cycle (Kamzolova and Morgunov 2019). In this process, citrate synthase, isocitrate dehydrogenase, and isocitrate lyase play important roles. Among them, citrate synthase participates in the synthesis of isocitric acid; while isocitrate dehydrogenase and isocitrate lyase are involved in the decomposition process of isocitric acid (Kamzolova and Morgunov 2019). It is worth noting that due to their low activity, these two key catalysts play a balancing role in maintaining normal metabolic pathways. The mitochondrial succinic acid-fumaric acid carrier YlSfc1 is the main control point of isocitric acid in *Y. lipolytica*. By overexpressing YlSfc1 and knocking out the citric acid transporter YlYHM2, the modified strain produced 136.7 g/L isocitric acid (Yuzbasheva et al., 2021). Overexpression of citric acid synthase *cit1* and *cit2* in *Y*. *lipolytica* significantly increased isocitric acid synthesis. Compared with the original strain, the synthesis of overexpressed *cit1* isocitrate increased by 9.5 times, and the synthesis of overexpressed *cit2* isocitrate increased by 6.8 times (Hapeta et al., 2020). By overexpressing glycerol kinase (*GUT1*) and glycerol-3-phosphate dehydrogenase (*GUT2*) in *Y*. *lipolytica*, 42.5 g/L isocitric acid was produced from crude glycerol (Rzechonek et al., 2019).

Further efforts in the research of citral production could include improving the strains that produce ICA, for example, by using genetic engineering techniques to modify and optimize the strains to enhance their efficiency and stability in producing ICA (Morgunov et al., 2020); at the same time, research on utilizing the diversity of microorganisms to screen for more suitable strains for ICA production could also be conducted (Kamzolova et al., 2021). In the fermentation process, in addition to optimizing the existing fermentation processes, exploring the introduction of new reactors or adjusting fermentation conditions to increase the yield and purity of ICA could also be pursued (Yuzbasheva et al., 2021).

Furthermore, using new inexpensive raw materials is an important direction, such as using agricultural waste and industrial by-products as sources of raw materials for ICA production to reduce costs and achieve sustainable development. Additionally, in the development of new methods for separating and purifying ICA (Schlembach et al., 2024), advanced separation techniques such as ultrafiltration and ion exchange chromatography should be combined with continuous exploration of innovative technologies to improve the quality and purity of ICA products (Kamzolova et al., 2016). In summary, there are still many worthwhile directions for further research and exploration in the field of ICA production.

**FIGURE 5:**
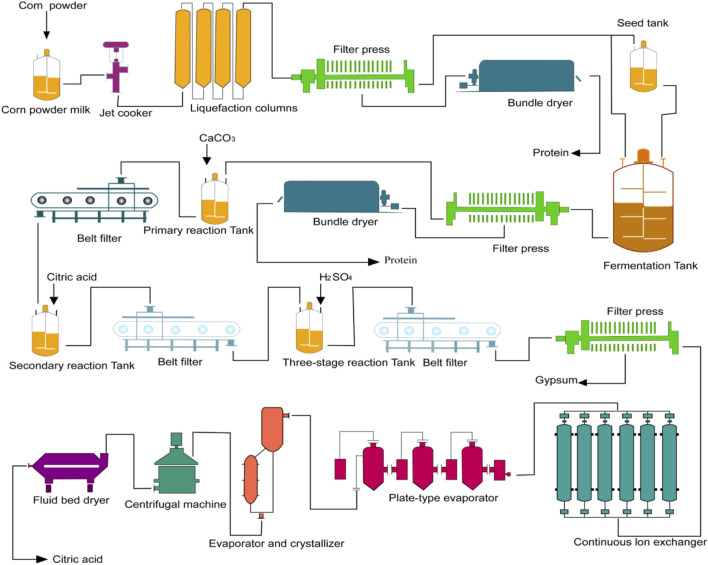
Citric acid production process. Including raw material treatment, culture, fermentation, mash treatment, extraction process, refining process, drying and packaging.

Currently, the most common method for biological fermentation of organic acids is the use of calcium salts. This method involves using CaCO_3_ to neutralize the organic acids in the fermentation liquid (Figure 5). The process includes several important steps such as fermentation, acid hydrolysis, purification, evaporation, crystallization, and drying. Acidolysis occurs when calcium isocitrate is used under acidic conditions with an increase in hydrogen ion concentration. In the presence of sulfuric acid, a double decomposition reaction takes place resulting in an insoluble gypsum precipitate and release of a weak acid (Mores et al., 2021). This reaction leads to irreversible precipitation of calcium sulfate (Figure 5). As a result, both consumed sulfuric acid and citric acid can be completely decomposed into citric acid and calcium sulfate (Kamzolova et al., 2013).

## 5 Summary

Metabolic engineering in microorganisms seeks to enhance the efficiency of biosynthetic routes, boost production yields, and reduce expenses in the manufacture of organic acids such as lactic acid, butyric acid, succinic acid, α-ketoglutaric acid, fumaric acid, citric acid, and isocitric acid. In the field of microbial metabolic engineering, efforts are directed towards enhancing biosynthetic pathways, improving production efficiency and cutting down on expenses in the manufacturing of lactic acid, butyric acid, succinic acid, α-ketoglutaric acid, fumaric acid, citric acid and isocitric acid.

Acid resistance is the core trait of industrial organic acid producing bacteria. The accumulation of organic acids leads to a sharp drop in pH, triggering an increase in intracellular H levels, undermining membrane potential, inhibiting enzyme activity and DNA stability. Acid-tolerant bacteria maintain pH homeostasis by enhancing proton efflux (e.g., *H-atpase*), synthesizing alkaline substances or modifying cell membranes, thereby reducing the cost of neutralizers, increasing product concentrations (e.g., *A. niger* produces citric acid at pH < 2.0) and inhibiting bacterial contamination (Hu et al., 2019).

Adaptive evolution (ALE) induces the accumulation of multigene mutations in strains through progressive pressurization (gradual reduction of pH) or dynamic stimulation (pH oscillations), including membrane structure strengthening, proton pump optimization, stress protein activation, and metabolic flux reprogramming. Combined with high-throughput screening and omics analysis, key genes can be located. Compared to metabolic engineering, ALE does not need to predict the target, but it takes a longer time. The collaborative strategy combines ALE base strains with CRISPR editing to accelerate tolerance optimization (Xu et al., 2025).

Methods for improving production efficiency focus on genetic modification of microbial strains, optimization of fermentation conditions, and the utilization of cost-effective raw materials. To sum up, the production and research of various organic acids are constantly advancing, improving production efficiency through various optimization measures, reducing costs, exploring new raw materials and new technologies, and achieving sustainable development.
